# Clinicopathologic analysis of renal cell carcinoma containing Intratumoral fat with and without osseous metaplasia

**DOI:** 10.1186/s13000-020-00941-z

**Published:** 2020-03-06

**Authors:** Zhaoying Xian, Jason O. Orien, Geoffrey N. Box, Debra L. Zynger

**Affiliations:** 1 Department of Pathology, The Ohio State University Medical Center, 410 W 10th Ave., 401 Doan Hall, Columbus, OH 43210 USA; 2 Department of Urology, The Ohio State University Medical Center, 410 W 10th Ave., 401 Doan Hall, Columbus, OH 43210 USA

**Keywords:** Renal cell carcinoma, Intratumoral fat, Osseous metaplasia, Angiomyolipoma

## Abstract

**Background:**

There is minimal information regarding the prevalence of intratumoral adipose in renal cell carcinoma (RCC), and no study has assessed the impact of intratumoral adipose on the preoperative imaging diagnosis. The aim of this study was to investigate the prevalence and histopathologic characteristics of entrapped adipose with or without osseous metaplasia in RCC nephrectomy specimens and to determine if this finding impacted the preoperative imaging interpretation.

**Methods:**

704 RCC specimens were prospectively evaluated for entrapped adipose and osseous metaplasia (423 partial nephrectomies, 281 total nephrectomies; 327 pT1a, 377 ≥ pT1b; 510 clear cell, 119 papillary, 30 chromophobe, 22 clear cell papillary, 23 other). Imaging reports were obtained, and the presence of intratumoral fat or calcification and the radiologic diagnostic impression were recorded.

**Results:**

3% (*n* = 21) contained microscopically identified intratumoral adipose, with a similar frequency in the main histologic subtypes (*p* = 0.76). Mean metaplastic deposit size was 0.4 cm, mean deposit to capsule distance 0.2 cm, and 29% involved the tumor capsule. Histologically identified adipose was infrequently noted via imaging (13%), and only 1 case with histologically identified metaplasia had a radiologic diagnostic differential of angiomyolipoma (1/704, 0.1%).

**Conclusion:**

While intratumoral adipose and/or osseous metaplasia can be observed within RCC, it is extremely rare for the radiologic diagnostic impression to have been confounded by histologically identified entrapped adipose. Awareness that metaplastic deposits are usually near the tumor capsule and may be minute could help prevent errors in diagnosis or staging.

## Background

Renal cell carcinoma (RCC), a malignant lesion and the most frequent kidney cancer found in adults, rarely contains intratumoral fat. Radiographic detection of fat within a renal mass is classically associated with angiomyolipoma (AML). This overlap potentially complicates the radiologic diagnosis of AML versus RCC and therefore may have a clinically significant impact as localized RCC is treated with surgical excision while AML may undergo surveillance without excision. Intratumoral adipose also may impact the pathologic pT classification and diagnosis of RCC [1]. If the adipose occurs at the periphery of the tumor and is misinterpreted as tumor invasion of perinephric fat, the tumor will be inaccurately classified as pT3a. Upon pathological analysis, intratumoral adipose within RCC has the potential to be misdiagnosed as AML, a common benign tumor of the kidney.

There is minimal information in the literature regarding the frequency of intratumoral adipose and osseous metaplasia in RCC [1]. No study has assessed the impact of intratumoral adipose on the preoperative radiographic imaging impression. The aim of this investigation is to determine the prevalence, impact on preoperative imaging, and histopathologic characteristics of intratumoral adipose and osseous metaplasia in RCC.

## Methods

### Study cohort

RCC nephrectomy cases with intratumoral adipose and/or osseous metaplasia at The Ohio State University Wexner Medical Center from 2010 to 2015, inclusive, were prospectively diagnosed with metaplasia documented by a single genitourinary pathologist (D.L.Z.) at the time of initial diagnosis. Adipose and osseous metaplasia documentation was performed as part of routine evaluation of RCC nephrectomy cases. Osseous metaplasia was defined as intratumoral calcification with the presence of lacunae and/or bone marrow elements. Other types of calcification (e.g. dystrophic calcification) did not meet our criteria and therefore were not included. Tumors were sampled at 1 block per cm of largest tumor diameter as per institutional protocol. The pathology report for each case was assessed to record the following information: RCC subtype, nephrectomy type (partial versus total), tumor size, grade, and pT classification. To minimize carryover artifact from biopsy and histologic processing, which can mimic intratumoral adipose without osseous metaplasia, only deposits adherent to the surrounding tissue were included. Small fragments of adipose tissue were not included. Additionally, no case had a prior needle core biopsy of the renal mass. All slides were analyzed to determine the size of the largest intratumoral focus of adipose and/or osseous metaplasia (in which adipocytes are often present as a part of the recapitulation of bone marrow) and the distance of the deposit from the nearest tumor edge/capsule. As fat cells are known to involve the tumor capsule or subcapsular space, only discrete metaplastic deposits were considered a positive identification. Radiographic imaging was performed using computed tomography (CT) and/or magnetic resonance imaging (MRI). Description of intratumoral fat or calcification in the preoperative radiographic imaging report and radiologic impression (RCC, AML) was recorded. Hounsfield units were measured for the mass and the area within the mass suspected to contain intratumoral adipose. Data was collected via an institutional review board-approved protocol.

### Statistical analysis

Statistical analyses were performed using unequal variances 2-tailed 2-sample t-testing and chi-square. As smaller tumors have a higher percentage of tumor sampled, tumors in the pT1a group were assessed separately and with the entire cohort to determine the frequency of intratumoral adipose and osseous metaplasia.

## Results

### Cohort

704 RCC surgical specimens were included, of which 423 were partial nephrectomies and 281 were total nephrectomies. 327 RCC cases were pT1a and 377 were ≥ pT1b. By RCC subtype, there were 510 clear cell, 119 papillary I/II, 30 chromophobe, 22 clear cell papillary, 8 unclassifiable, 7 multilocular cystic, 3 translocation, and 5 other.

The mean age for RCC cases that contained intratumoral adipose and/or osseous metaplasia was 62.0 years. 19% (*n* = 4) of cases were female and 81% (*n* = 17) were male. There was no statistical difference in mean age, median tumor grade, frequency of intratumoral adipose or osseous metaplasia, size of the largest focus, distance of the nearest focus to the tumor edge/capsule, frequency of fat or calcification on radiographic imaging, or rate of inclusion of AML in the radiographic differential diagnosis for partial versus total nephrectomy cases. As expected, tumors resected by total nephrectomy had a larger mean tumor size (8.7 cm versus 3.5 cm, *p* = 0.04). There was no difference in the frequency of intratumoral adipose and/or osseous metaplasia for pT1a versus ≥pT1b (*p* = 0.15).

### Cases with intratumoral adipose and/or osseous metaplasia

**Fig. 1:**
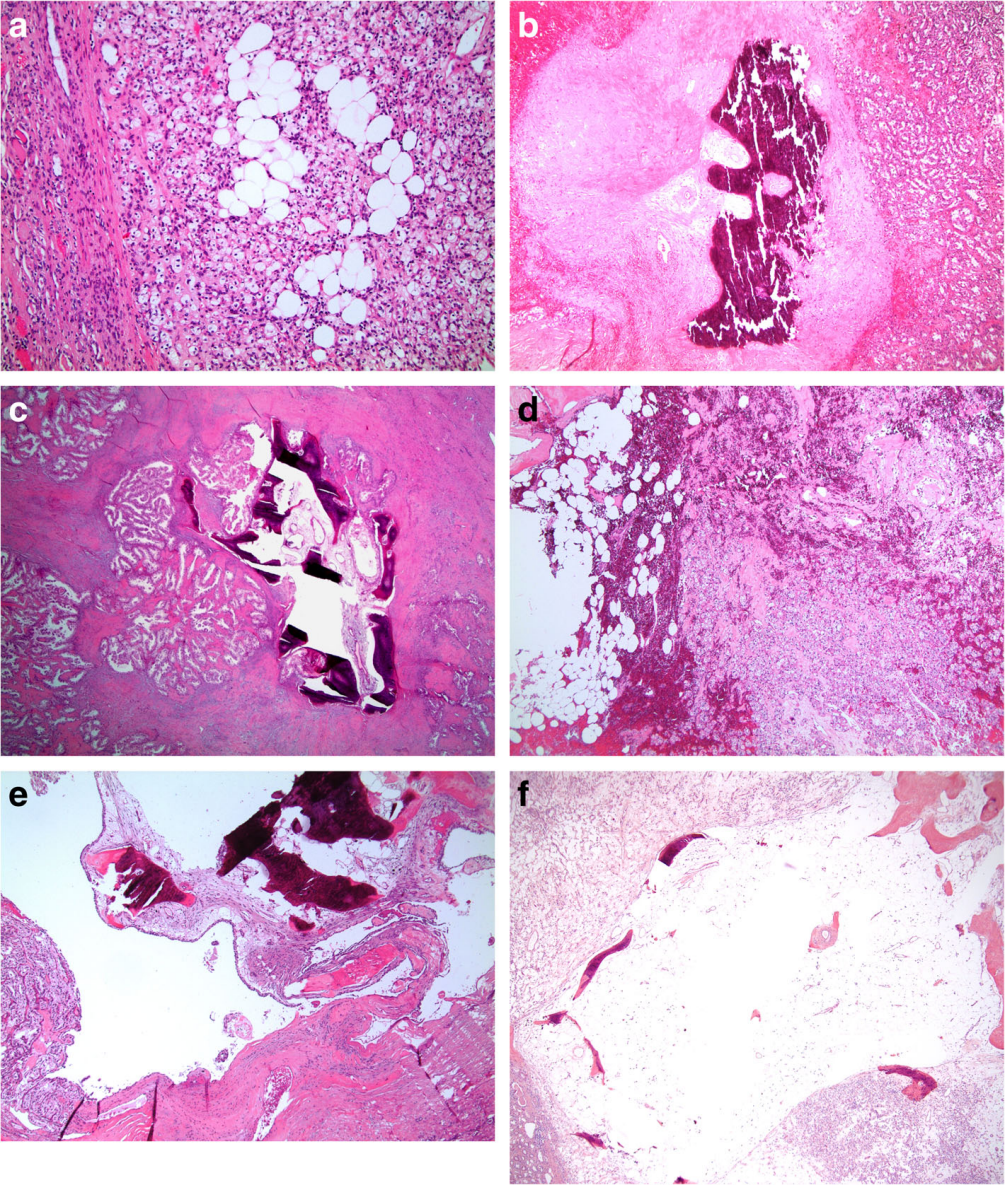
Photomicrographs of renal cell carcinoma (RCC) with adipose and/or osseous metaplasia. **a,** RCC, clear cell type with a minute (0.1 cm) deposit of adipose metaplasia near the tumor edge (Case 14 on Table 2) (10X magnification). **b,** RCC, papillary type I with osseous metaplasia consisting of mostly bone and only rare adipocytes (Case 11 on Table 2) (4X magnification). **c**, RCC with leiomyomatous-like stroma containing a focus of osseous metaplasia with occasional adipocytes (Case 12 on Table 2) (2X magnification). **d**, RCC, chromophobe type with adipose metaplastic deposit located centrally within the tumor (Case 17 on Table 2) (4X magnification). **e,** RCC, clear cell papillary type with osseous metaplasia containing adipose adjacent to the tumor capsule (Case 10 on Table 2) (2X magnification). **f,** RCC, clear cell type with osseous metaplasia consisting of predominantly adipocytes abutting the tumor edge, AML was considered in the radiologic differential diagnosis for this case (Case 1 on Table 2) (4X magnification)

Clinicopathologic and radiologic characteristics of cases with intratumoral adipose and/or osseous metaplasia are depicted in Table 1. 3% (*n* = 21) of the cohort contained intratumoral adipose and/or osseous metaplasia (3% of partial nephrectomy cases and 2% of radical nephrectomy) (Table 2, Fig. 1). 2% (*n* = 13) of cases contained adipose with osseous metaplasia (fat with bone), 1% (*n* = 4) of cases contained adipose without associated osseous metaplasia (fat without bone), and 1% (n = 4) of cases contained osseous metaplasia without associated adipose (bone without fat). The mean metaplastic deposit size was 0.4 cm (range 0.1–1.3 cm). Metaplastic deposits had a mean distance to the tumor edge/capsule of 0.2 cm (range 0–0.8 cm) with 29% involving the tumor capsule (Fig. 1e, f). A similar frequency of deposits was observed within each subtype of RCC (clear cell, 3%; papillary I/II, 4%; chromophobe, 3%; and clear cell papillary, 5%; *p* = 0.76).

**Table 1:** Demographic, pathologic, and radiographic characteristics

				Pathologic Findings							Radiologic Findings		
Case	Sex/Age (y)	RCC type	Surgery	Tumor Size, (cm)	Grade	pT	Size of Largest Focus (cm)	Distance to Capsule (cm)	Adipose	OM	Fat	Ca^2+^	AML Considered
1	F/55	CC	P	3.3	2	1a	0.5	0	Y	Y	N	N	Y
2	M/56	CC	P	1.9	2	1a	0.3	0.3	Y	Y	N	N	N
3	M/54	CC	P	3.3	2	1a	0.4	0.04	Y	Y	N	N	N
4	M/65	CC	P	1.7	3	1a	0.8	0	Y	Y	N	N	N
5	M/52	CC	P	3.9	1	1a	0.8	0.4	Y	Y	N	N	N
6	F/70	CC	P	2.7	3	1a	0.4	0.5	Y	Y	N	N	N
7	M/66	CC	T	12.5	3	3a	0.2	0.8	Y	Y	N	N	N
8	M/74	CC	P	4.2	4	3a	1.3	0.1	Y	Y	N	Y	N
9	M/51	CC	P	5.7	3	1b	0.5	0	Y	Y	N	Y	N
10	M/52	CCP	T	2.5	2	1a	0.3	0.1	Y	Y	N	Y	N
11	M/77	Pap I	T	4	2	1a	0.4	0	Y	Y	N	Y	N
12	M/53	RCC, LMLS	P	1.7	3	1a	0.4	0.2	Y	Y	N	Y	N
13	M/69	Pap II	P	5.8	2	1b	0.3	0.4	Y	Y	Y	Y	N
14	M/69	CC	T	2.4	2	1a	0.1	0.03	Y	N	N	N	N
15	F/54	CC	T	6.8	3	1b	0.3	0.3	Y	N	N	N	N
16	F/58	CC	P	3.4	3	1a	0.2	0	Y	N	N	N	N
17	M/74	CH	T	5.8	3	3a	0.5	0.4	Y	N	N	N	N
18	M/79	CC	P	3.7	3	1a	0.2	0	N	Y	N	N	N
19	M/65	Pap I	P	2.8	3	1a	0.3	0.2	N	Y	N	N	N
20	M/71	Pap II	P	4.2	3	1b	0.2	0.4	N	Y	Y	Y	N
21	M/38	Pap I & II	T	26.8	3	2b	0.4	0.5	N	Y	NA	NA	NA

*AML*, angiomyolipoma; *Ca*^*2+*^, calcification; *CC*, clear cell; *CCP*, clear cell papillary; *CH*, chromophobe; *F*, female; *LMLS*, leiomyomatous-like stroma; *M*, male; *N*, No; *NA*, data not available; *OM*, osseous metaplasia; *Pap I*, papillary type 1; *Pap II*, papillary type 2; *P*, partial nephrectomy; *RCC*, renal cell carcinoma; *T*, total nephrectomy; *Y*, Yes

**Table 2:** Clinicopathologic and radiologic findings

**Pathologic Findings**	
Median Grade	3
Mean Tumor Size (cm)	5.2
Intratumoral Adipose Only	4/21 (19%)
Osseous Metaplasia Only	4/21 (19%)
Adipose and Osseous Metaplasia	13/21 (62%)
Mean Metaplastic Deposit Size (cm)	0.4 (range 0.1–1.3)
Mean Deposit to Capsule Distance (cm)	0.2 (range 0–0.8)
**Radiologic Findings**	
Fat	2/20 (10%)
Calcification	7/20 (35%)
AML Considered	1/20 (5%)

*AML*, angiomyolipoma

Radiology reports were available for 20 of 21 cases with deposits. Histologically identified intratumoral adipose, observed in 17 of the cases with radiology reports, was infrequently detected via imaging (*n* = 2, 12%) and raised the differential diagnosis of AML in 1 case as described below. Histologically identified osseous metaplasia, observed in 16 of the cases with radiology reports, was more frequently noted via imaging (*n* = 7, 44%) (*p* = 0.04) and in no case was the radiologic differential diagnosis of AML raised.

### Frequency of AML in the differential diagnosis of cases with histologically identified metaplasia

Only 1 case with histologically identified metaplasia had a radiologic diagnostic differential of AML (1/704, 0.1%) (Fig. 1f). Enhancement on CT was seen in a 3.6 cm lesion at the anterior mid aspect of the kidney that was thought to possibly represent AML. However, RCC could not be excluded. The enhanced attenuation of the whole mass and of the area of suspected intratumoral adipose were 86 and 25 Hounsfield units, respectively. The patient subsequently had an MRI in which the mass demonstrated heterogeneous decreased T1 and heterogeneous increased T2 signal intensity, and positive gadolinium enhancement. The enhancing solid mass was noted to lack intratumoral fat and was highly suspicious for RCC. AML was not included in the MRI radiologic differential diagnosis. On pathologic analysis, this case contained intratumoral adipose with osseous metaplasia, and it was diagnosed as RCC, clear cell type.

## Discussion

Radiographic evidence of fat within a renal mass can be seen in both AML and RCC, potentially complicating the radiographic diagnosis of AML versus RCC. Intratumoral adipose in RCC is usually associated with osseous metaplasia [1–14], but AML is also known to rarely present with calcifications [15–20]. Furthermore, RCC has been reported to contain adipose without evidence of calcification on CT and/or pathologic analysis [3, 7–9, 14].

In RCC in which intratumoral adipose is present, the adipose is often associated with osseous metaplasia, defined as bone matrix with associated osteoblasts, osteoclasts, adipocytes, and/or hematopoietic cells [1–14]. The exact etiology of intratumoral adipose is unknown, but there are three recognized mechanisms by which RCC can contain fatty elements: 1) entrapment of perinephric fat by tumor growth, 2) presence of lipid vacuoles and cholesterol clefts due to necrosis, and 3) development of fatty marrow elements within areas of osseous metaplasia [1–8]. When associated with osseous metaplasia, the intratumoral fat is thought to be secondary to a metaplastic process rather than due to invasion of perinephric fat or necrosis [1].

We identified intratumoral adipose and/or osseous metaplasia in 3% of RCC cases. A single previous study investigated the prevalence of intratumoral adipose within RCC with a stated incidence of 0.3% [1], but the calculation was based on retrospectively reviewed reports without a central review of slides over a wide time period (23 years, 1987–2009). In contrast, our cases were diagnosed by a single pathologist with the metaplasia prospectively documented at the time of the initial diagnosis over a narrower period (6 years, 2010–2015). This difference in methodology may have yielded a lower incidence in the prior report. As anticipated, histologically identified osseous metaplasia was radiologically detected more frequently than histologically identified intratumoral adipose (44% versus 12%) in our cohort, similar to the prior study (67 and 18%, respectively) [1].

Previously, intratumoral adipose with or without osseous metaplasia has been documented in a few clear cell, papillary I/II, and chromophobe RCC cases [1–14]. We demonstrated that the frequency of adipose metaplasia was similar between different subtypes of RCC. Additionally, intratumoral fat in clear cell papillary RCC and leiomyomatous-like stroma RCC subtypes are identified for the first time. Of note, RCC with leiomyomatous-like stroma is known to demonstrate smooth muscle proliferation that extends into perinephric fat, which can be confused for adipose metaplasia; however, the epithelial component usually does not, thus allowing for differentiation between the two distinct entities [21].

Tumor size (pT1a versus ≥pT1b) did not impact incidence of adipose metaplasia. Intratumoral adipose has been described more commonly at the periphery within RCC [1, 2, 4, 5, 7, 8, 10, 11, 14]. Our findings corroborate this notion in that we found the mean adipose to capsule distance to be 0.2 cm, with 29% of metaplastic deposits involving the tumor capsule. Clinicians therefore should be aware that adipose or calcifications at the tumor edge may not be indicative of AML or fat invasion. A prior mean size of largest focus of intratumoral adipose was reported to be 1.0 cm [1], larger than the mean size detected in our cohort (0.4 cm). As mentioned, our study prospectively documented metaplasia and thus may have been more likely to include smaller deposits.

Only 0.1% of our RCC cases with histologically identified metaplasia had a radiologic diagnostic differential of AML by CT. A few case reports of fat-containing RCC have described how the presence of intratumoral fat on CT led to the erroneous diagnosis of AML [2, 3, 5, 8, 10, 14], but no prior investigation has assessed the frequency with which intratumoral adipose in RCC raised the differential diagnosis of AML. While we identified a single case in which the CT was concerning for AML, the MRI findings were consistent with RCC. MRI has greater tissue sensitivity and previously has been shown to be effective for the differential diagnosis of renal masses that are inconclusive on CT or ultrasound [22]. Therefore, when a renal mass is non-diagnostic on CT, clinicians can consider further workup with to MRI help distinguish between RCC vs AML.

## Conclusion

Our study demonstrated that intratumoral adipose and/or osseous metaplasia occur more frequently (3%) than previously described, with a similar prevalence in the main histologic subtypes of RCC including clear cell, papillary I/II, chromophobe, and clear cell papillary tumors. Histologically identified intratumoral adipose and/or osseous metaplasia is seen on imaging in only a subset of cases, and it is extremely rare (0.1%) for microscopically identified metaplasia to have confounded the radiologic diagnosis by mimicking AML. When the radiologic diagnosis is uncertain, further evaluation with MRI can help clarify the diagnosis. However, physicians should be aware that adipose and/or osseous metaplasia occur in RCC, and that the deposits are usually near the tumor edge and may be minute, in order to avoid diagnostic or staging errors.

## Data Availability

Data and materials of this work are available from the corresponding author on reasonable request.

## Abbreviations

AML: Angiomyolipoma
Ca^2+^: Calcification
CC: Clear cell
CCP: Clear cell papillary
CH: Chromophobe
CT: Computed tomography
F: Female
LMLS: Leiomyomatous-like stroma
M: Male
MRI: Magnetic resonance imaging
N: No
NA: Data not available
OM: Osseous metaplasia
P: Partial nephrectomy
Pap I: Papillary type 1
Pap II: Papillary type 2
RCC: Renal cell carcinoma
T: Total nephrectomy
Y: Yes
